# The Effect of Timing of Female Vibrational Reply on Male Signalling and Searching Behaviour in the Leafhopper *Aphrodes makarovi*

**DOI:** 10.1371/journal.pone.0139020

**Published:** 2015-10-21

**Authors:** Anka Kuhelj, Maarten de Groot, Andrej Blejec, Meta Virant-Doberlet

**Affiliations:** Department of Entomology, National Institute of Biology, Ljubljana, Slovenia; San Diego, UNITED STATES

## Abstract

Sexual communication in animals often involves duetting characterized by a coordinated reciprocal exchange of acoustic signals. We used playback experiments to study the role of timing of a female reply in the species-specific duet structure in the leafhopper *Aphrodes makarovi* (Hemiptera: Cicadellidae). In leafhoppers, mate recognition and location is mediated exclusively by species- and sex-specific substrate-borne vibrational signals and a female signal emitted in reply to male advertisement calls is essential for recognition and successful location of the female. In *A*. *makarovi*, males have to initiate each exchange of vibrational signals between partners, and in a duet the beginning of a female reply overlaps the end of the male advertisement call. Results of playback treatments in which female replies were delayed and did not overlap with the male call revealed that in order to trigger an appropriate behavioural response of the male, female reply has to appear in a period less than 400 ms after the end of the initiating male call. Results also suggest that males are not able to detect a female reply while calling, since female reply that did not continue after the end of male call triggered male behaviour similar to behaviour observed in the absence of female reply. Together, our results show that vibrational duets are tightly coordinated and that the species-specific duet structure plays an important role in mate recognition in location processes.

## Introduction

Communication enables sexual partners to recognize and find each other and is an essential part of reproductive behaviour [1]. Sexual communication based on acoustic signals often involves reciprocal exchange of signals between partners [2, 3]. The coordinated exchange of air-borne and substrate-borne acoustic signals is usually termed duetting and has been described in many arthropod and vertebrate taxa [2–5]. Duets are characterized by a predictable and stereotyped timing of signals and temporal coordination is expressed in reply latency, as well as in alternation or overlapping of signals [2, 3, 6]. In comparison with birds [3, 7, 8], duetting in insects has received much less attention [2], most likely because the exchange of signals between partners is not typical in insects communicating with air-borne sounds [9, 10]. However, in the last decade it became obvious that the most common and taxonomically widespread form of acoustic communication in insects is signalling by substrate-borne vibrations [11–14], where partners usually establish a duet [2, 15].

Despite the increasing awareness of the importance of vibrational signalling in animal communication, the structure of vibrational duets has only rarely been systematically studied. In the lacewings of the *Chrysoperla carnea* group (Chrysopidae, Neuroptera) males and females exchange sexually monomorphic species-specific vibrational signals [16]. Partners closely match their response latencies and appropriately timed responses are important in sex recognition [17] and in maintaining reproductive isolation [18]. The exchange of vibrational signals has a stereotyped, species-specific temporal pattern also in other insects relying on vibrational duetting; although, in these groups signals are also sex-specific [19–22]. Besides mate recognition and mate choice [15], the function of a duet has also been associated with localization of a partner [23–26]. Although recent studies suggest that in leafhoppers a precisely coordinated exchange of male and female vibrational signals is essential for mate recognition and successful location of the female [25, 26], the role of species-specific duet structure in mate searching has not been studied so far. Leafhoppers (Hemiptera, Cicadellidae) are one of the most speciose groups of phytophagus insects, with more than 22 000 species [27] and more detailed studies on such diverse group should improve our understanding of mechanisms underlying evolution of duetting behaviour.

**Fig 1:**
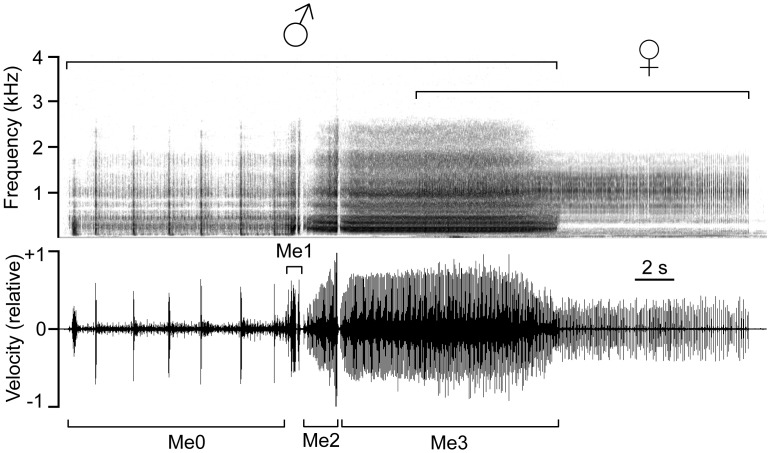
Representative male-female duet in *A*. *makarovi*. Spectrogram (above) and waveform (below) are shown. Me0-Me3: species-specific elements as described in [25].

Mate searching includes displays to attract and/or to advertise to a potential mate (i.e. emission of advertisement calls), as well as mobility needed to increase the signalling space and/or to locate the partner [28]. In the present study, we investigated the effect of timing of a female reply on male calling and searching behaviour in the leafhopper *Aphrodes makarovi*. As in other leafhoppers, mate recognition and location in this species is mediated exclusively via substrate-borne signals [29–31]. Males use the ‘fly/jump/walk-call’ strategy to increase their signalling space [32, 33]. Sexual communication is based on a duet initiated by a long and complex male advertisement call to which a sexually receptive virgin female responds (Fig 1), thus triggering the male search for the female on the plant [31]. Female reply is essential for successful mating, since male does not approach the female if she does not respond. Previous research showed that female replies with temporal parameters outside species-specific values had negative effect on recognition, as well as on male’s ability to locate the source of female vibrational signal [30]. Furthermore, the duet structure also suggests the existence of a species-specific time window of female reply. In this species, the beginning of a female reply overlaps the end of the male call [31] and although the onset of a female reply in *A*. *makarovi* is variable, the temporal association between the male call and the female response is nevertheless stereotyped and predictable. The onset of female vibrational reply before the conclusion of the initiating male call has been observed also in other insect taxa relying on vibrational communication [6, 20–22, 34, 35]. The critical response time window associated with the recognition of a female reply and triggering of locomotion related to searching behaviour is characteristic for air-borne sound duets in phaneropterine bushcrickets [36–39]; however, in vibrational communication systems its presence has been so far suggested only in alder flies from the genus *Sialis* (Neuroptera) [40] and in the psyllid *Diaphorina citri* [41].

A duet structure in which a female reply overlaps with his call presents the male with a fundamental problem of detecting the female reply while calling [42]. In *A*. *makarovi* vibrational signals are generated by a tymbal-like mechanism on the first abdominal segment [43, 44] and are transmitted to the substrate via the legs in which vibration receptors are also located [45, 46]. In this species male call and female reply are both composed of pulses (Fig 1) and there is no difference in the pulse repetition time between the last section of the male call and the female reply [29, 31]. Furthermore, the amplitude of female reply is always below the amplitude of male call [31].

We hypothesized (1) that a delayed female reply that does not overlap with the end of male call and, consequently, does not continue immediately after the end of male call, has a negative effect on male mate searching behaviour and (2) that males are not able to detect a female reply while emitting advertisement calls. To test these hypotheses we used playback experiments and we assessed male signalling and searching behaviour in response to female replies applied with different timing in relation to the male call. We predicted (a) that in the presence of a delayed female reply that does not appear immediately after the end of the call, males’ success of finding the female would be reduced (i.e. associated with lower calling rate and lower number of males localizing the source) and (b) that in the presence of a hidden reply that does not continue after the end of the call, male behaviour would be similar to behaviour in the absence of a female reply.

## Materials and Methods

### Insect collection and maintenance

The study does not involve endangered or protected species and no specific permits were required. Last stage nymphs and adults of *A*. *makarovi* were collected using a sweep net from alfalfa (*Medicago sativa*) and stinging nettle (*Urtica dioica*) at various localities in Slovenia (46° 03.259’N, 14° 27.719’ E; 46° 15.287’N, 13° 28.073’ E; 45° 27.300’N, 13° 42.078’ E) that are not environmentally protected or privately owned. In the laboratory males and females were kept separately in plastic boxes (38 x 26 x 27 cm) at 23–28°C, 50–70% humidity and 15: 9 h (L: D) photoperiod. They were fed with cut alfalfa placed in vials filled with water and replaced twice a week. Due to morphological similarities between *Aphrodes* species, the species identity of leafhoppers used in behavioural tests was determined prior to the experiments by recording their vibrational signals [29]. Males used in behavioural trials were 3–4 weeks old and were put individually in plastic cups (volume 0.5 L) a day before the start of experiments.

### Experimental set-up

**Fig 2:**
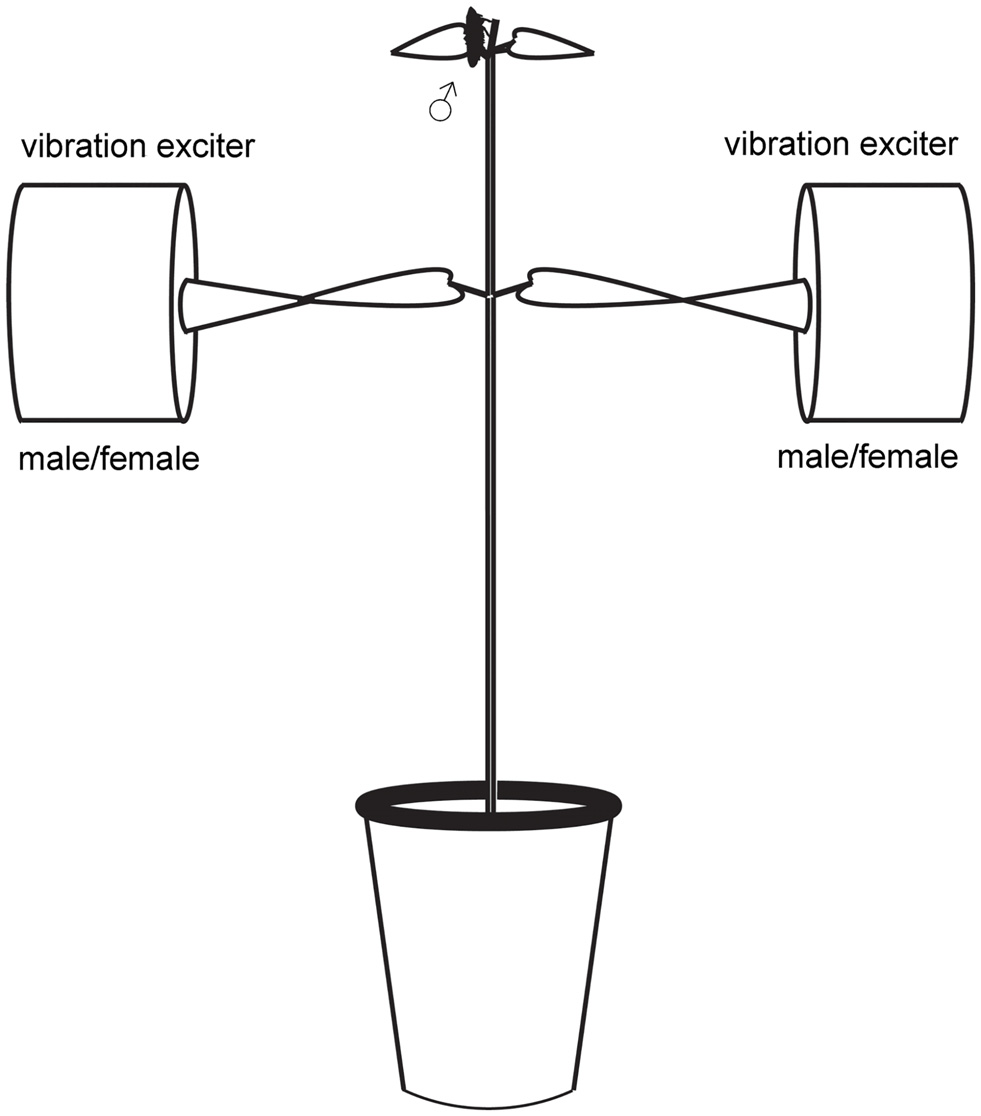
Experimental set-up. A schematic drawing of the experimental setup showing the initial position of the male on the top of the nettle plant and the positions of vibration exciters (not drawn to scale).

For all behavioural tests we used experimental set-up that enabled bilateral playback stimulation and was described previously [30, 31] (Fig 2). All experiments were performed on a stinging nettle (*U*. *dioica*) at room temperature (20–25°C) and 40–50% relative humidity. The top of the plant (height approximately 25 cm) was cut off and the bottom of the stem was inserted into a vial filled with water (to prevent withering) and placed upright into a jar filled with moist artificial substrate. All inflorescences and leaves, except the pair at the apex and another pair approximately 12 cm down the stem, were removed. Each of the lower leaves was attached to a separate vibration exciter (see below) and a new plant was taken every second day. To prevent males from escaping, a rectangular cage (65 x 65 x 50 cm) without the front panel was put over the plant.

We applied vibrational stimuli (described below) to the lower nettle leaves via the conical tip of the 5-cm metal rod (4 mm in diameter) screwed firmly into the head of a vibration exciter (Minishaker type 4810, Brüel & Kjær, Nærum, Denmark) (Fig 2) and fixed to the tip of the leaf with Blu-tack. Vibration exciter was driven from the computer via Sound Blaster X-Fi Surround 5.1 pro sound card (Creative, Singapore) by Cool Edit Pro2 (Syntrillium Software, Phoenix, USA). Transmission of vibrations through the plant results in degradation of the signals due to frequency filtering [47–49]. To assure the fidelity of signals perceived by males, we checked each day before starting the playback experiments, as well as several times throughout each experimental day, that the frequency characteristics of playback signals recorded on the plant correspond to the input signal by comparing spectral properties of the applied and recorded female reply. Stimulatory and emitted vibrational signals were registered from the reflective tape placed on the nettle stem 1 cm below the branching point of the lower leaves with a laser vibrometer (PDV-100, Polytech, GmbH, Waldbronn, Germany) and stored in a computer using the above mentioned sound card and Cool Edit Pro2 software at the sampling rate 48-kHz and 16-bit resolution. Male behaviour together with recorded vibrational signals was simultaneously filmed with a 3CCD video camcorder (Canon DM XM2).

### Experimental protocol and vibrational stimuli

Males of *A*. *makarovi* were tested in playback experiments in which we simulated a female response. A single male was placed on the leaves at the apex of the nettle plant (Fig 2). In order to induce the emission of advertisement calls we played him once a pre-recorded duet [30, 50]. We randomly applied to one of the lower leaves a male advertisement call while the other was vibrated with a female reply in order to simulate a male-female duet. Afterwards, whenever the live male emitted an advertisement call, we presented him with a female reply. The application of a female reply was triggered manually [30, 31, 50] (see below). The amplitude of stimulation was adjusted to the level of naturally emitted male advertisement calls and female replies registered at the point of recording (male call: 0.3 mm/s, female reply: 0.2 mm/s). The side from which we applied the female reply changed randomly. In control trials in the absence of female reply (see below) we observed male behaviour for 15 minutes. The trials in which we simulated the female reply continued for 15 minutes after stimulation with a duet or until the male localized the vibration exciter, whichever came first.

We tested males in two experimental series that each included 14 males. In both series males were tested with the same seven playback treatments in which we varied the timing of the female reply (see below). In each series we also included the F_0_ control treatment in which males were stimulated once with a duet to induce calling; however, afterwards they received no female replies to their calls emitted during the trial. Males were tested only once per day and each male was tested once with each treatment and their order was randomized for each male. The female signal used in playback experiments was chosen from the signal library at the National Institute of Biology and was composed of natural pulses assembled by the use of Cool Edit Pro 2 computer program [30]. The pulse repetition time (52 ms) corresponded to the mean values determined in previous studies [29, 31] and such composed female reply triggers the same male signalling and searching behaviour as observed in the natural male-female duets [31].

To assess the effect of a delay, i.e. time between the end of male call and the beginning of female reply on mate searching effort, males were tested in five treatments (Table 1) in which they were presented with a 10.4 s long female reply that corresponds to the mean non-overlapped value determined previously [31]. In the F_10_ treatment we simulated a natural duet by timing the onset of the female replies with the typical decrease in the amplitude in the last section (Me3) of the male advertisement call (Fig 1), so that the beginning of the female reply overlapped the end of male call as in a natural *A*. *makarovi* duet. In the other four treatments, the female replies were delayed for 0.4, 0.8, 1.5 or 2 s after the end of male call (Table 1). In these four treatments, the onset of a playback of a female reply, which in these cases incorporated also the specified reply delay, was timed with the end of male call. Due to unpredictable variations in the duration of the Me3 section and errors in manual triggering of a playback, the actual reply delays varied somewhat; however, the delay ranges did not overlap among treatments (F_10+400_: 406 ± 42 ms (mean ± SD); F_10+800_: 816± 57 ms; F_10+1500_: 1503 ± 57 ms; F_10+2000_: 2020 ± 57 ms).

**Table 1:** Temporal characteristics (duration and timing) of *A*. *makarovi* female replies used in playback experiments. Hypothesis 1: A delayed female reply has a negative effect on male mate searching behaviour. Hypothesis 2: Males are not able to detect a female reply while emitting advertisement calls. 0: natural timing of female reply (the beginning of female reply overlapping the end of male call). +: onset of female reply delayed for 400–2000 ms after the end of male call (female reply not overlapping with male call). H: female reply hidden in the last section of male call (female reply completely overlapping with the last section of male call).

	Treatment	Duration (s)	Timing
**Hypothesis 1**	**F_10_** *	10.4	0
	**F_10+400_**	10.4	+400
	**F_10+800_**	10.4	+800
	**F_10+1500_**	10.4	+1500
	**F_10+2000_**	10.4	+2000
**Hypotheis 2**	**F_5_**	5.2	0
	**F_5H_**	5.2	H

*: F_10_ treatment represents a naturally timed female reply of average duration

To test whether males perceive a female reply during emission of advertisement calls, males were presented with 5.2 s female replies and the onset of the female responses was timed with the beginning of the Me3 section so that the female signals remained completely hidden in the male calls (Table 1). For these tests we chose a shorter-than-average female reply within the natural range determined previously [29, 31], since the duration of the Me3 section is often shorter than 10.4 s [31]. In the F_5_ treatment, we simulated a natural duet in which only the beginning of such short female replies overlapped with the end of male calls (Table 1).

In all experiments we monitored the following parameters: number of advertisement calls emitted by males, calling rate (number of calls emitted per min of trial after a duet was established), duration of emitted male calls, number of males searching (searching was defined as leaving the apex of the nettle plant and walking during and/or immediately after female reply), number of males locating the source and searching time (time needed to locate the source after the onset of searching).

### Statistical analyses

**Fig 3:**
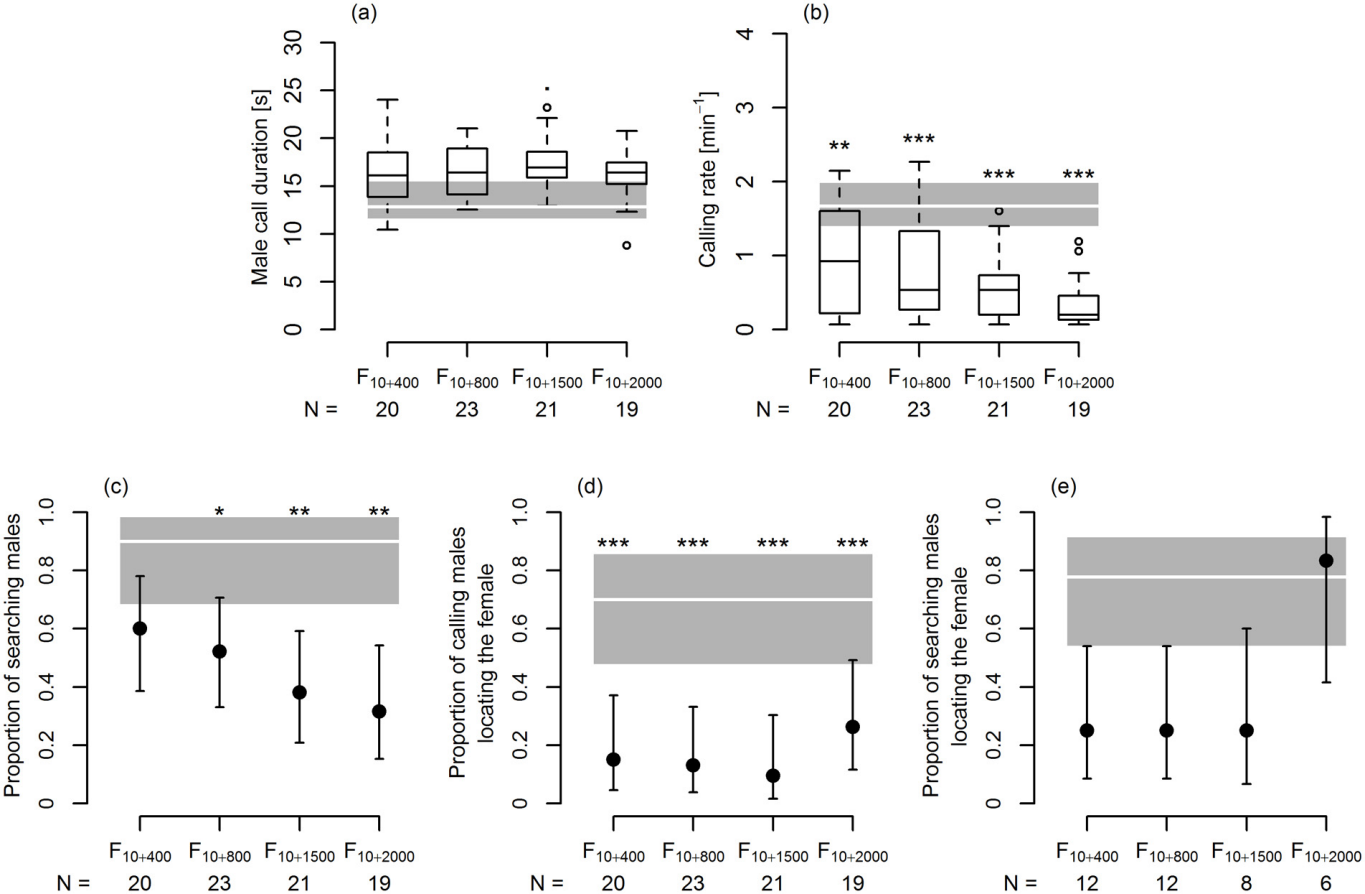
The effect of delay of female reply on signalling and searching behaviour of *A*. *makarovi* males. (a) male advertisement call duration; (b) calling rate; (c) proportion of males searching for the source of female reply; (d) proportion of calling males locating the source; (e) proportion of searching males locating the source. (a, b) box and whisker plots show the median (black line), the 25–75% interquartile range (boxes), the lowest and the highest data points still within 1.5 of interquartile range (whiskers) and outliers (circles). Values obtained in the F_10_ treatment are shown as median (thick white line) together with 95% confidence interval for median (gray area). *, ** and *** indicate significant difference from the F_10_ treatment (Lme model followed by Dunnett’s multiple comparisons test, p < 0.05, p < 0.01 and p < 0.001, respectively). (c-e) determined proportion (black circle) together with 95% confidence interval for proportions is shown. Proportion obtained in the F_10_ treatment (thick white line) is shown together with 95% confidence interval for proportions (gray area). *, ** and *** indicate values that are significantly lower than in the F_10_ treatment (Regwq multiple comparisons test, p < 0.05, p < 0.01 and p < 0.001, respectively). N = number of males included in the analyses. F_10_: N = 20 (a-d), N = 18 (e).

**Fig 4:**
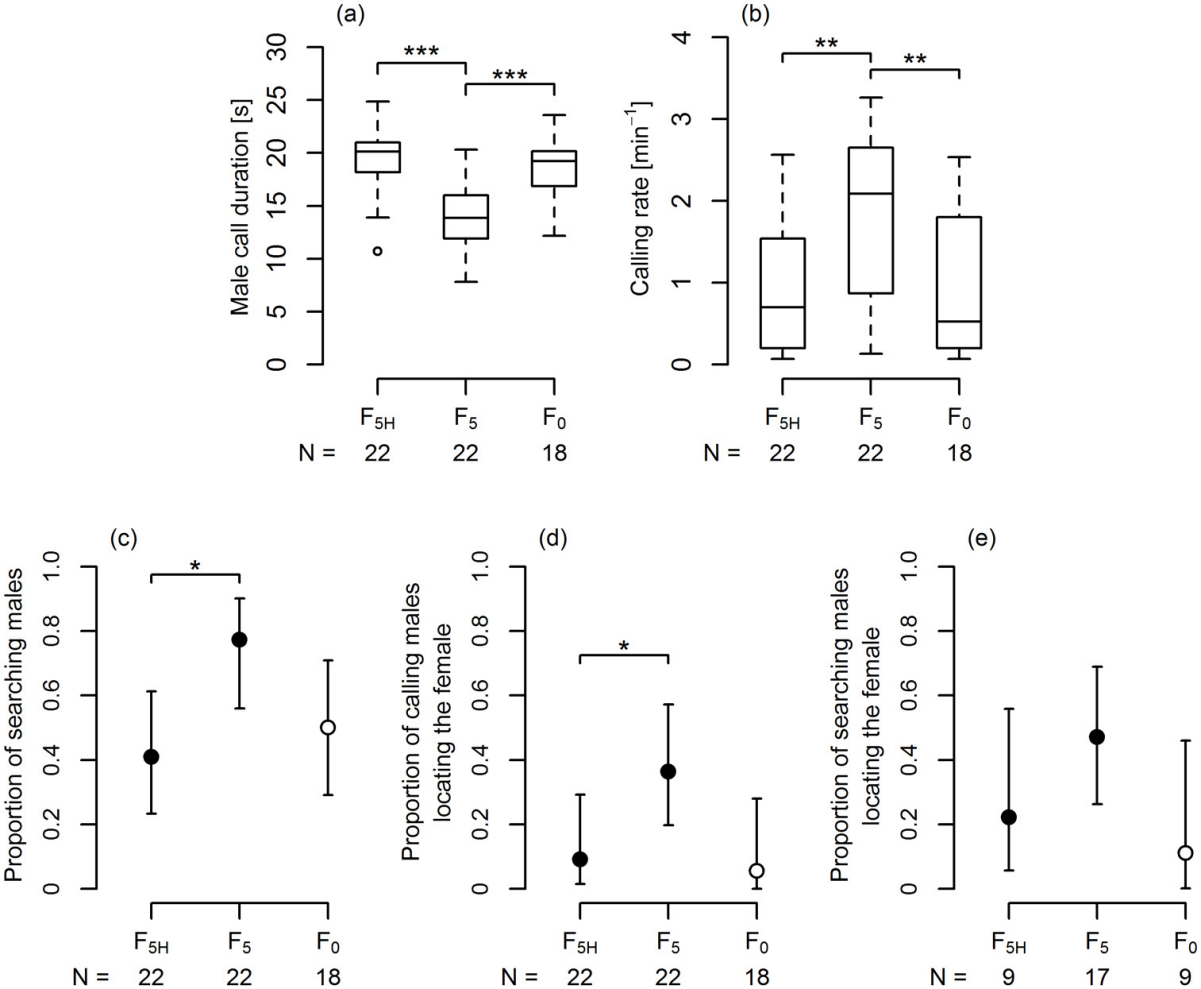
The effect of hidden female reply on signalling and searching behaviour of *A*. *makarovi* males. (a) male advertisement call duration; (b) calling rate; (c) proportion of males searching for the source of female reply; (d) proportion of calling males locating the source; (e) proportion of searching males locating the source. (a, b) box and whisker plots show the median (black line), the 25–75% interquartile range (boxes), the lowest and the highest data points still within 1.5 of interquartile range (whiskers) and outliers (circles). *, ** and *** indicate significant difference between treatments (Lme model followed by Tukey’s all pair comparisons test, p < 0.001). (c-e) determined proportion (black circle) together with 95% confidence interval for proportions is shown. * indicate values that are significantly lower than in the F5 treatment (one-tailed Fisher’s exact test, p < 0.05). Values obtained in the F_0_ treatment (white circles) shown for comparison indicate the number of males changing their position during the trial (c) and the number of males arriving to the leaves (d, e) and were not included in statistical analyses.

We included in the analyses only active males that emitted advertisement calls (i.e. calling males) and the numbers of males used in the analyses for each treatment are shown in Figs 3a and 4a for treatments with delayed and hidden replies, respectively. To analyse the success in locating the source of female reply, we took into account the number of calling males, as well as the number of males that were also searching for the female on the plant (i. e. searching males). Only males that located the source of female reply were included in the analyses of searching time.

To assess the effect of a delay of female reply on male signalling behaviour, we compared the values obtained in four treatments in which the female reply was delayed with the values obtained in the F_10_ treatment (naturally timed, i.e. partly overlapped female reply of average duration) using a linear mixed-effect (Lme) model in which male identity was included as a random effect [51, 52] followed by Dunnett’s multiple comparisons test with a control [53]. The Ryan-Einot-Gabriel-Welsch (Regwq) multiple comparisons test was used to compare the numbers of searching males and numbers of males locating the source [54].

To test whether males perceive a female reply during emission of advertisement calls, calling parameters obtained in the treatment with a hidden female reply (F_5H_) were compared with values obtained in the F_5_ and F_0_ treatments using the above mentioned Lme model followed by Tukey’s all pair comparisons test [53]. One-tailed Fisher’s exact test was used to compare the numbers of searching males and numbers of males locating the source in the F_5_ and F_5H_ treatments. Numbers obtained in the F_0_ treatment were not included in these analyses, since in the absence of female reply, leaving the apex of the plant is associated with random movements and cannot be characterized as searching behaviour.

Due to the small numbers of males locating the source in most treatments (see Results), a meaningful statistical comparison of searching time was possible only for the F_10_ and F_10+2000_ treatments. The values obtained in these two treatments were compared using the Wilcoxon signed rank test.

All statistical analyses were conducted using R version 3.2.0 [55].

## Results

### The effect of a female reply delay on male signalling and searching behaviour

In comparison with the F_10_ treatment, in which males received the naturally timed (i.e. partly overlapped) female reply, the duration of male advertisement call was significantly longer when female replies were delayed for 800, 1500 and 2000 ms (Fig 3a). Furthermore, the calling rate was significantly lower in all four treatments with delayed female replies (Fig 3b). The number of males searching for the female was significantly reduced in correlation with the increasing reply delay (Fig 3c). Moreover, taking into account the number of calling males, in all treatments with the delayed female response, the number of males locating the source of female reply was significantly lower than when the beginning of female reply overlapped the end of male call (Fig 3d). Although taking into account only males searching for the female, in the F_10+2000_ treatment higher proportion of searching males located the source than in other treatments with a delayed reply, the numbers of males locating the source in all treatments with delayed replies were not significantly lower than in the F_10_ treatment, probably owing to the small number of males searching for the female in some treatments (Fig 3e). Due to the small numbers of males locating the source of the delayed female replies a meaningful statistical comparison of searching time was possible only for the F_10+2000_ treatment, in which searching time did not differ significantly from the F_10_ treatment (S1 Fig). Taken together, these results support the existence of male sensory time window immediately after the end of male advertisement call.

### Perception of female reply during calling

When female replies did not continue after the end of male call (F_5H_ treatment), the average male call duration was significantly longer than in the treatment with partly overlapping female replies of the same duration (F_5_ treatment); however, it did not differ significantly from the F_0_ control treatment in which males did not receive any reply (Fig 4a). Moreover, also the calling rate in the F_5H_ treatment did not differ from the calling rate in the F_0_ treatment, while it was significantly lower than in the F_5_ treatment (Fig 4b). Significantly fewer males searched for the source of a hidden reply than for the source of a partly overlapped female reply of the same duration (Fig 4c) and only two males arrived at the vibrated leaf in this treatment. In the F_0_ treatment, 50% of males moved from the starting position at the apex of the nettle plant during the trial; however, only one of these males walked to the leaf in the allotted time. Taking into account the number of calling males, the number of males locating the source in the F_5H_ treatment was significantly lower than in the treatment with partly overlapping female reply of the same duration (Fig 4d). However, owing to the small number of males searching for the female, the difference between these two treatments was not significant, when only searching males had been taken into account. Since only two males located the source in the F_5H_ treatment, a meaningful statistical comparison of searching time was not possible (S1 Fig). Taken together, these results suggest that males did not detect female reply during emission of advertisement calls.

## Discussion

Results of the present study show that in *A*. *makarovi* the timing of a female reply has profound effects on male signalling and searching behaviour and influenced the likelihood of finding the female. Our results indicate that in this species males are not able to detect female reply while calling; however, in order to trigger the appropriate mate searching behaviour female reply has to appear in the time window immediately after the end of male call.

As in other vibrational communication systems in which mate searching has been studied in more detail [24, 26, 32, 33, 56], in *A*. *makarovi* female reply is essential in triggering searching behaviour, as well as for successful and quick location of the female; however, males have to initiate each exchange of vibrational signals. Males of many small plant-dwelling insects relying on vibrational communication use ‘fly/jump/walk-call’ strategy to increase their signalling space [25, 32, 33, 57]. Such strategy, however, presents males with a fundamental problem of reliably detecting a replying conspecific female, since her presence in male’s local environment is usually unpredictable. It has been hypothesized that a predictable temporal association between the initiating call and the reply signal may be advantageous in communication between partners since it enables mate recognition in the environment with high levels of abiotic and biotic noise [3, 36, 37, 58]. For plant dwelling-arthropods, behaviourally relevant abiotic noise arises predominantly from wind [59–61]. Furthermore, incidental interference from heterospecific signals is common in acoustic communication [62] and individuals of different species living on the same plant can emit vibrational signals simultaneously [12]. *Aphrodes makarovi* leafhoppers communicate in a complex biotic landscape that contains other heterospecific signallers and includes also congeners living syntopically [29] and therefore incidental interference that may result in false alarms is likely. False alarms (i.e. responding to a heterospecific female) are costly due to wasted effort and unnecessary exposure to risk and may ultimately also result in mating with heterospecifics [63]. In *A*. *makarovi* vibrational signalling incurs direct costs due to eavesdropping predators [64], as well indirect costs due to high energy expenditure, and males with higher calling effort die sooner [50]. Males of this species show high selectivity in their behavioural response to female reply. It should be noted that the number of males arriving at the vibration exciter in most treatments with delayed female replies corresponds to the number of males randomly walking to the leaf in the absence of female reply.

The results of the present study show that *A*. *makarovi* male has to detect a female reply in a period less than 400 ms after the end of his call in order to respond appropriately. It has been shown in another species in this genus (*A*. *bicincta*) that males can contribute more than females to sexual isolation between species and that male’s contribution to assortative mating resulted from mate recognition as well as from inability to locate the source of an inappropriate female reply due to a breakdown of a species-specific duet structure [25]. Although a duet structure in *A*. *makarovi* superficially appears simpler than in *A*. *bicincta*, in which female replies have to appear in short intervals between continuously repeated elements in male call [25], the results obtained in the present study support the view that vibrational duets are tightly coordinated and that the species-specific duet structure plays an important role in maintaining reproductive isolation.

After they perceive a reply from a conspecific female, males of *A*. *makarovi* increase their calling rate and commence searching on a plant [30, 31, 50] and therefore calling rate and triggering of locomotion associated with searching for the female could be linked with mate recognition process. However, male has to stimulate each emission of female vibrational signal and as in other duetting systems [15, 24, 65] in *A*. *makarovi* higher calling rate also increases a probability of locating the female [50]. Although with current data it is not possible to determine to what extent a female reply outside the male’s sensory time window selectively affects recognition and/or localization processes, some of our results suggest that different time delays may affect these two processes differently. While 400 and 800 ms time delays appear to have proportionally larger effect on localization, those *A*. *makarovi* males that were searching in the treatment with the longest female reply delay were highly successful in locating the source regardless of their low calling rate. The success of males locating the source of delayed replies in this treatment may be attributed either to higher motivation or better location ability [66]. These males may also show better behavioural plasticity in switching to alternative tactics, such as satellite behaviour (i.e. silent approach to a female duetting with another male) [57, 67, 68]. Since in a natural situation *A*. *makarovi* males never overlap their calls further studies should also determine the effect of female reply with a delay that takes into account a duration of rival’s call (i.e. delays longer than 10 s).

Calling and listening to other acoustic signals in the environment presents signallers with a fundamental sensory problem of discriminating between their own (i.e. self-generated) and environmental (i.e. external) signals [42]. While insects communicating with air-borne sounds are able to detect external sounds while singing [69, 70], results of the present study indicate that *A*. *makarovi* male is not able to perceive a female reply during emission of an advertisement call. It should be noted that in this species isolated males emit longer advertisement calls than males duetting with the female [30, 31]. In duetting bushcrickets, when male air-borne call is long and complex, female reply is usually triggered by a particular sound element indicating the end of male call [58, 71–73]. It has also been shown that female reply is triggered by specific elements in vibrational male advertisement calls [21, 35]. In *A*. *makarovi* the duration of the last section in the call is long and highly variable (between 5–20 s) [31] and currently there is no evidence that the timing of a female reply is correlated with various components of male call. Consequently, by replying before the male call is finished female should maximize the chance that the reply appears in the male’s sensory time window [39], especially if the position of male’s recognition sensory time window is immediately after the end of his call. While in duetting bushcrickets the sensory time window is often narrow (20–50 ms) [36–38], the suggested time windows in both vibrational communication systems studied so far were wider (400–500 ms) [40, 41]. The lack of detailed information on temporal coordination of duets in other vibrational communication systems precludes more generalized comparisons; however, it is conceivable that due to the relatively low propagation velocities of vibrational signals in plants [47, 74, 75] the resulting transmission time delays are large in comparison with air-borne sound and, consequently, sensory time windows should be wider. Results obtained in the present study suggest that the sensory time window of *A*. *makarovi* males may be shorter than 400 ms; however, in order to determine the exact position and width of the time window, a development of an automated system, which would reliably distinguish male vibrational calls from background noise and trigger playback of female replies in real-time with short time delay, is needed. Such system would be invaluable also for testing other species in this genus in which observed female reply delays in natural duets are around 50 ms [76].

Our results suggest that male of *A*. *makarovi* perceives only that part of a female reply that continues after the end of his call. However, walking associated with searching behaviour is also limited to the duration of non-overlapped female reply and to a period shortly afterwards [30, 31] and leg movements stimulate the same leg mechanoreceptors that also detect vibrational signals. There is no direct information to what extent walking influences perception of female reply; however, it should be noted that males need on average around 1 s after the end of the call to commence searching [30, 31]. Neuronal mechanisms underlying vibrational communication are virtually unexplored [75, 77] and more elaborate behavioural and neurophysiological studies are needed to unravel mechanisms underlying processes of mate recognition and directionality in insects communicating via substrate-borne vibrational signals.

The importance of female signals in the mate recognition process and sender-receiver dynamics is becoming increasingly clear [78] and the results of the present study show that *A*. *makarovi* provides a good opportunity to explore the importance of female signals in a duet structure. In this species, the duration of a female reply is long and variable [29, 31] and males locate the source of longer female replies faster [30]. Additional studies should elucidate whether observed variation in female reply is related to mate choice [15]. However, males are able to detect and evaluate only the non-overlapped section of female reply, which depends not only on the latency and overall duration of female reply, but also on duration of the initiating male call itself. Further studies should also determine whether males adjust their signalling behaviour according to the duration of female reply in order to optimize their mate searching behaviour.

## Supporting Information

S1 Fig The effect of timing of female reply on searching time of *Aphrodes makarovi* males. (a) treatments with a delayed female response; (b) treatment with a hidden female reply. Raw data are shown. (b) Value obtained in the F_0_ treatment (white circle) shown for comparison indicates the time male needed to arrive to the leaf. N = number of trials included in the analyses.
(PDF)

S1 Table Raw data from playback treatments used to assess the effect of female reply delay. The table summarizes signalling and searching behavioural parameters for each male scored in each treatment.
(PDF)

S2 Table Raw data from playback treatments used to assess whether males perceive female reply while calling. The table summarizes signalling and searching behavioural parameters for each male scored in each treatment.
(PDF)

## References

[1] Shuster SM, Wade MJ. Mating systems and mating strategies. Princeton: Princeton University Press; 2003.

[2] Bailey WJ. Insect duets: underlying mechanisms and their evolution. Physiol Entomol. 2003;28: 157–174.

[3] Hall ML. A review of vocal duetting in birds. Adv Stud Behav. 2009;40: 67–121.

[4] Emerson SB, Boyd SK. (1999). Mating vocalizations of female frogs: control and evolutionary mechanisms. Brain Behav Evolut. 1999;53: 187–197. 10.1159/000006594 10343085

[5] Uhl G, Elias DO. Communication In: Heberstein ME, editor. Spider behaviour: flexibility and versatility. Cambridge: Cambridge University Press; 2011 pp. 128–189.

[6] Boumans L, Johnsen A. Stonefly duets: vibrational sexual mimicry can explain complex patterns. J Ethol. 2015;33: 87–107.

[7] Hall ML. A review of hypotheses for the functions of avian duetting. Behav Ecol Sociobiol. 2004;55: 415–430.

[8] Todt D, Naguib M. Vocal intercations in birds; the use of song as a model in communication. Adv Stud Behav. 2000;29: 247–296.

[9] Cooley JR, Marshall DC. Sexual signalling in periodical cicadas *Magicicada* spp. (Hemiptera: Cicadidae). Behaviour. 2001;138: 827–855.

[10] Robinson DJ, Hall MJ. Sound signalling in Orthoptera. Adv Insect Physiol. 2002;29: 151–278.

[11] Virant-Doberlet M, Čokl A. Vibrational communication in insects. Neotrop Entomol. 2004;33: 121–134.

[12] Cocroft RB, Rodríguez RL. The behavioral ecology of insect vibrational communication. BioScience. 2005;55: 323–334.

[13] Hill PSM. Vibrational communication in animals. Cambridge, MA: Harvard University Press; 2008.

[14] Cocroft RB, Gogala M, Hill PSM, Wessel A. Fostering research progress in a rapidly growing field In: Studying vibrational communication. Berlin, Heidelberg: Springer-Verlag; 2014 pp. 3–12.

[15] Rodríguez RL, Barbosa F. Mutual behavioral adjustment in vibrational duetting In: Studying vibrational communication. Berlin, Heidelberg: Springer-Verlag; 2014 pp. 147–169.

[16] Henry CS, Brooks SJ, Duelli P, Johnson JB, Wells MM, Mochizuki A. Obligatory duetting behaviour in the *Chrysoperla carnea*-group of cryptic species (Neuroptera: Chrysopidae): its role in shaping evolutionary history. Biol Rev. 2013;88: 787–808. 10.1111/brv.12027 23433087

[17] Henry CS, Wells MLM. Sexually dimorphic intrasexual duetting in an otherwise monomorphic green lacewing (Neuroptera, Chrysopidae, *Chrysoperla plorabunda*): sexual selection or sex recognition. J Insect Behav. 2009;22: 289–312.

[18] Henry CS, Wells MLM. Testing the ability of males and females to respond to altered songs in the duetting green lacewing *Chrysoperla plorabunda* (Neuroptera: Chrysopidae). Behav Ecol Sociobiol. 2006;61: 39–51.

[19] Tishechkin D. Y. Vibrational communication in Aphrodinae leafhoppers (Deltocephalinae auct. Homoptera: Cicadellidae). Russian Entomol J. 2000;9: 1–66.

[20] Percy DM, Taylor GS, Kennedy M. Psyllid communication: acoustic diversity, mate recognition and phylogenetic signal. Invertebr Syst. 2006;20: 431–445.

[21] Rodríguez RL, Cocroft RB. Divergence in female duetting signals in the *Enchenopa binotata* species complex of treehoppers (Hemiptera: Membracidae). Ethology. 2006;112: 1231–1238.

[22] Stewart KW, Sandberg JB. Vibratory communication and mate searching behaviour in stoneflies In: Insect sounds and communication: physiology, behaviour, ecology and evolution. Boca Raton, Florida: Taylor & Francis; 2006 pp. 179–186.

[23] Abbot JC, Stewart KW. Male search behavior of the stonefly *Pteronarcella badia* (Hagen) (Plecoptera: Pteronarcyidae) in relation to drumming. J Insect Behav. 1993;6: 467–481.

[24] Legendre F, Marting PR, Cocroft RB. Competitive masking of vibrational signals during mate-searching in a treehopper. Anim Behav. 2012;83: 361–368.

[25] Derlink M, Pavlovčič P, Stewart AJA, Virant-Doberlet M. Mate recognition in duetting species: the role of male and female vibrational signals. Anim Behav. 2014;90: 181–193.

[26] Polajnar J, Eriksson A, Stacconi Rossi MV, Lucchi A, Anfora G, Virant-Doberlet M, et al The process of pair formation mediated by substrate-born vibrations in small insect. Behav. Processes. 2014;107: 68–78. 10.1016/j.beproc.2014.07.013 25101559

[27] Dietrch CH. Phylogeny of the leafhopper Family Evacanthinae with a review of Neotropical species and notes on related groups (Hemipetra; Membracoidea; Cicadellidae). Syst Entomol. 2004;29: 455–487.

[28] Kokko H, Wong BBM. What determines sex roles in mate searching? Evolution. 2007;61: 1162–1175. 17492969 10.1111/j.1558-5646.2007.00090.x

[29] Bluemel JK, Derlink M, Pavlovčič P, Russo I-RM, King RA, Corbett E, et al Integrating vibrational signals, mitochondrial DNA and morphology for species determination in the genus *Aphrodes* (Hemiptera: Cicadellidae). Syst Entomol. 2014;39: 304–324.

[30] de Groot M, Čokl A, Virant-Doberlet M. Searching behaviour in two hemipteran species using vibrational communication. Centr Eur J Biol. 2011;6: 756–769.

[31] de Groot M, Derlink M, Pavlovčič P, Prešern J, Čokl A, Virant-Doberlet M. Duetting behaviour in the leafhopper *Aphrodes makarovi* (Hemiptera: Cicadellidae). J Insect Behav. 2012;25: 419–440.

[32] Hunt RE, Nault LR. Roles of interplant movement, acoustic communication and phototaxis in mate-location behavior of the leafhopper *Graminella nigrifrons* . Behav Ecol Sociobiol. 1991;28: 315–320.

[33] De Luca PA, Cocroft RB. The influence of age on male mate-searching behaviour in thornbug treehoppers. Ethology. 2011;117: 1–11.

[34] de Vrijer PWF. Variability in calling signals of the planthopper *Javesella pellucida* (F.) (Homoptera: Delphacidae) in relation to temperature and consequences for species recognition during distant communication. Neth J Zool. 1984;34: 388–406.

[35] Hunt RE, Fox JP, Haynes KF. Behavioral response of *Graminella nigrifrons* (Homoptera; Cicadellidae) to experimentally manipulated vibrational signals. J Insect Behav. 1992;5: 1–13.

[36] Heller KG, von Helversen D. Acoustic communication in phaneropterid bushcrickets: species-specific delay of female stridulatory response and matching male sensory time window. Behav Ecol Sociobiol. 1986;18: 189–198.

[37] Robinson D, Rheinlaender J, Hartley JC. Temporal parameters of male-female sound communication in *Leptophytes punctatissima* . Physiol Entomol. 1986;11: 317–323.

[38] Zimmermann U, Rheinlaender J, Robinson D. Cues for male phonotaxis in the duetting bushcrisket *Leptophytes punctatissima* . J Comp Physiol. 1989;164: 621–628.

[39] Bailey WJ, Hammond TJ. Female reply stregies in a duetting Australian bushcricket *Caedicia* sp. (Phaneropterinae: Tettigoniidae: Orthoptera). J Exp Biol. 2004;207: 803–811. 14747412 10.1242/jeb.00840

[40] Rupprecht R. Die Kommunikation von *Sialis* (Megaloptera) durch Vibrationssignale. J Insect Physiol. 1975;21: 305–320.

[41] Rohde B, Paris TM, Heatherington EM, Hall DG, Mankin RW. Responses of *Diaphorina citri* (Hemiptera: Psyllidae) to conspecific vibrational signals and synthetic mimics. Ann Entomol Soc Am. 2013;106: 392–399.

[42] Hoy R. Tuning by turning off. Nature. 2002;48: 831–832. 10.1038/418831a 12192395

[43] Ossiannilsson F. Insect drummers. A study on the morphology and function of the sound-producing organ of Swedish Homoptera Auchenorrhyncha with notes on their sound-production. Opuscula Entomol Suppl. 1949;X: 1–145.

[44] Pringle JWS. The structure and evolution of the organs sound-production in cicadas. Proc Linn Soc. 1957;167: 144–159.

[45] Čokl A, Virant-Doberlet M, Zorović M. Sense organs involved in the vibratory communication of bugs In: Insect sounds and communication: physiology, behaviour, ecology and evolution. Boca Raton, Florida: Taylor & Francis; 2006 pp. 71–80.

[46] Lakes-Harlan R, Strauss J. Functional morphology and evolutionary diversity of vibration receptors in insects In: Studying vibrational communication. Berlin, Heidelberg: Springer-Verlag; 2014 pp. 277–302.

[47] Michelsen A, Fink F, Gogala M, Traue D. Plants as transmission channel for insect vibrational songs. Behav Ecol Sociobiol. 1982;1: 269–281.

[48] Cocroft RB, Shugart HJ, Konrad KT, Tibbs K. Variation in plant substrates and its consequences for insect vibrational communication. Ethology. 2006;112: 779–789.

[49] Cocroft RB, Hamel J, Su Q, Gibson J. Vibrational playback experiments In: Studying vibrational communication. Berlin, Heidelberg: Springer-Verlag; 2014 pp. 249–274.

[50] Kuhelj A, De Groot M, Pajk F, Simčič T, Virant-Doberlet M. Energetic cost of vibrational signalling in a leafhopper. Behav Ecol Sociobiol. 2015;69: 815–828.

[51] Laird NM, Ware JH. Random-effects models for longitudinal data. Biometrics. 1982;38: 963–974. 7168798

[52] Davidian M., Giltinan DM. Nonlinear Models for Repeated Measurement Data. Boca Raton, FL, USA: Chapman & Hall/CRC; 1995.

[53] Thorsten H, Bretz F, Westfall P. Simultaneous inference in general parametric models. Biometr J. 2008;50: 346–363. 10.1002/bimj.200810425 18481363

[54] Hochberg Y, Tamhane JC. Multiple Comparison Procedure. 1987 New York, NY, USA: Wiley; 1987.

[55] R Development Core Team. A language and environment for statistical computing, R Foundation for Statistical Computing, Vienna; 2015.

[56] De Winter AJ, Rollenhagen T. The importance of male and female acoustic behaviour for reproductive isolation in *Ribautodelphax* planthoppers (Homoptera: Delphacidae). Biol J Linn Soc. 1990;40: 191–206.

[57] Mazzoni V, Prešern J, Lucchi A, Virant-Doberlet M. Reproductive strategy of the Nearctic leafhopper *Scaphoideus titanus* Ball (Hemiptera: Cicadellidae). Bull Entomol Res. 2009;99: 401–413. 10.1017/S0007485308006408 18947453

[58] Dobler S, Heller K-G, von Helversen O. Song pattern recognition and an auditory time window in the female bushcricket *Ancistrura nigrovitata* (Orthoptera: Phaneropteridae). J Comp Physiol A. 1994;175: 67–74.

[59] Barth FG, Bleckmann H, Bohnenberger J, Seyfarth E-A. Spiders of genus *Cupiennius* Simon 1891 (Araneae, Ctenidae). II. On the vibratory environment of a wandering spider. Oecologia. 1988;77: 194–201. 28310372 10.1007/BF00379186

[60] Tishechkin DY. Background noises in vibratory communication channels of Homoptera (Cicadinea and Psyllinea). Russian Entomol J. 2007;16: 39–46.

[61] McNett GD, Luan LH, Cocroft RB. Wind-induced noise alters signaller and receiver behaviour in vibrational communication. Behav Ecol Sociobiol. 2010;64: 2043–2051.

[62] Gröning J, Hochkirch A. Reproductive interference between animal species. Q Rev Biol. 2008;83, 257–282. 18792662 10.1086/590510

[63] Virant-Doberlet M, Mazzoni V, De Groot M, Polajnar J, Lucchi A, Symondson WOC. et al. Vibrational communication networks: eavesdropping and biotic noise In: Studying vibrational communication. Berlin, Heidelberg: Springer-Verlag; 2014 pp. 93–123.

[64] Virant-Doberlet M, King RA, Polajnar J, Symondson WOC. Molecular diagnostics reveal spiders that exploit vibrational signals used in sexual communication. Mol Ecol. 2011;20: 2204–2216. 10.1111/j.1365-294X.2011.05038.x 21352388

[65] Rodríguez RL, Sullivan LE, Cocroft RB. Vibrational communication and reproductive isolation in the *Enchenopa binotata* species complex of treehoppers (Hemiptera: Membracidae). Evolution. 2004;58: 571–578. 15119440

[66] Kokko H, Rankin DJ. Lonely hearts or sex in the city? Density dependent effects in mating systems. Phil Trans R Soc B. 2006;361: 319–334. 16612890 10.1098/rstb.2005.1784 PMC1569612

[67] Mazzoni V, Lucchi A, Čokl A, Prešern J, Virant-Doberlet M. Disruption of reproductive behaviour of *Scaphoideus titanus* by playback of vibrational signals. Entomol Exp Appl. 2009;133: 174–185.

[68] Kuhelj A. Sexual competitors in the communication strategy of the southern green stink bug (*Nezara viridula*, Pentatomidae) and the leafhoppers of the genus *Aphrodes* (Cicadellidae). Ph.D. Thesis, University of Ljubljana. 2015.

[69] Hennig RM, Weber T, Huber F, Kleindienst H.-U, Moore TE, Popov AV. Auditory threshold change in singing cicadas. J Exp Biol. 1994;187: 45–55. 9317307 10.1242/jeb.187.1.45

[70] Poulet JFA, Hedwig B. A corollary discharge maintains auditory sensitivity during sound production. Nature. 2002;418: 872–876. 12192409 10.1038/nature00919

[71] Heller K-G. Evolution of song pattern in east Mediterranean Phaneropterinae: constrains by the communication system In: Bailey WJ, Rentz DCF, editors. The Tettigoniidae: biology, systematics and evolution. Bathurst: Crawford House Press; 1990 pp. 130–151.

[72] Bailey WJ, Field G. Acoustic satellite behaviour in the Australian bushcricket *Elephantodeta nobilis* (Phaneropterinae, Tettigoniidae, Orthoptera). Anim Behav. 2000;59: 361–369. 10675258 10.1006/anbe.1999.1325

[73] Stumpner A, Meyer S. Songs and the function of song elements in four duetting bushcricket species (Ensifera, Phaneropteridae, *Barbistes*). J Insect Behav. 2001;14: 511–534.

[74] Čokl A, Zorović M, Millar JC. Vibrational communication along plants by stink bugs *Nezara viridula* and *Murgantia histrionica* . Behav Processes. 2007;75: 40–54. 17306471 10.1016/j.beproc.2007.01.003

[75] Virant-Doberlet M, Čokl A, Zorović M. Use of substrate vibrations for orientation: from behaviour to physiology In: Insect sounds and communication: physiology, behaviour, ecology and evolution. Boca Raton, Florida: Taylor & Francis; 2006 pp. 81–97.

[76] Derlink M. (2014). Vibrational signals, reproductive isolation and speciation in the genus *Aphrodes* Curtis, 1883 (Hemiptera: Cicadellidae). Ph.D. Thesis, University of Ljubljana. 2014.

[77] Zorović M. Temporal processing of vibratory communication signals at the level of ascending interneurons in *Nezara viridula* (Hemiptera: Pentatomidae). PLoS One, 2011;6: e26843 10.1371/journal.pone.0026843 22053216 PMC3203904

[78] Wilkins MR, Seddon N, Safran RJ. Evolutionary divergence in acoustic signals: causes and consequences. Trends Ecol Evol. 2013;28: 156–166. 10.1016/j.tree.2012.10.002 23141110

