# Supplementary material for: The Effect of Timing of Female Vibrational Reply on Male Signalling and Searching Behaviour in the Leafhopper Aphrodes makarovi
Source: PLoS One. 2015 Oct 21;10(10):e0139020. doi: 10.1371/journal.pone.0139020 (PMC4619402; doi:10.1371/journal.pone.0139020)
Supplement: S1 Fig — (a) treatments with a delayed female response; (b) treatment with a hidden female reply. Raw data are shown. (b) Value obtained in the F0 treatment (white circle) shown for comparison indicates the time male needed to arrive to the leaf. N = number of trials included in the analyses. (PDF) [file pone.0139020.s001.pdf]

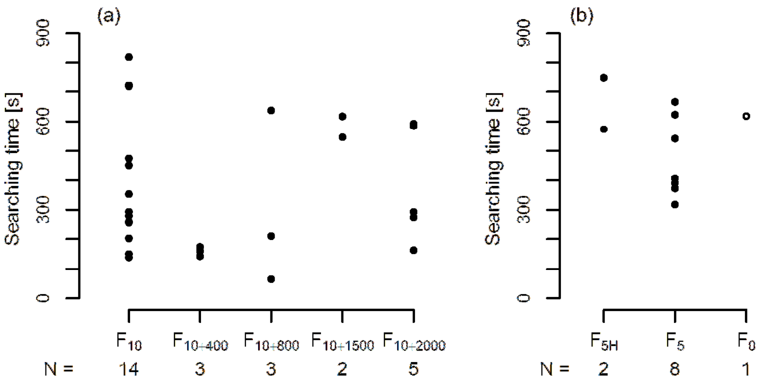

S1 Figure. The effect of timing of female reply on searching time of *Aphrodes makarovi* males. (a) treatments with a delayed female response; (b) treatment with a hidden female reply. Raw data are shown. (b) Value obtained in the F_0 treatment (white circle) shown for comparison indicates the time male needed to arrive to the leaf. N = number of trials included in the analyses.
