# Supplementary material for: The Effect of Timing of Female Vibrational Reply on Male Signalling and Searching Behaviour in the Leafhopper Aphrodes makarovi
Source: PLoS One. 2015 Oct 21;10(10):e0139020. doi: 10.1371/journal.pone.0139020 (PMC4619402; doi:10.1371/journal.pone.0139020)
Supplement: S1 Table — The table summarizes signalling and searching behavioural parameters for each male scored in each treatment. (PDF) [file pone.0139020.s002.pdf]

S2 Table. Raw data from playback treatments used to assess the effect of female reply delay. The table summarizes signalling and searching behavioural parameters for each male scored in each treatment.

male_identity	experiment	number of calls	average call duration [s]	calling rate [min^{-1}]	searching	locating	searching time [s]
1	F_{10} (control)	12	9,626	1,951	1	1	260
2	F_{10} (control)	NA	NA	NA	NA	NA	NA
3	F_{10} (control)	25	17,260	1,667	1	0	NA
4	F_{10} (control)	18	12,797	1,982	1	1	451
5	F_{10} (control)	NA	NA	NA	NA	NA	NA
6	F_{10} (control)	11	11,843	1,646	1	1	255
7	F_{10} (control)	14	11,300	1,503	1	1	474
8	F_{10} (control)	10	15,457	0,667	0	NA	NA
9	F_{10} (control)	33	16,340	2,200	1	0	NA
10	F_{10} (control)	13	13,024	2,155	1	1	280
11	F_{10} (control)	7	21,741	1,368	1	1	149
12	F_{10} (control)	NA	NA	NA	NA	NA	NA
13	F_{10} (control)	10	14,420	2,247	1	1	202
14	F_{10} (control)	33	12,836	2,526	1	1	718
15	F_{10} (control)	13	12,795	2,047	1	1	293
16	F_{10} (control)	NA	NA	NA	NA	NA	NA
17	F_{10} (control)	NA	NA	NA	NA	NA	NA
18	F_{10} (control)	22	20,613	1,467	0	NA	NA
19	F_{10} (control)	16	12,824	1,067	1	0	NA
20	F_{10} (control)	19	7,928	1,287	1	1	818
21	F_{10} (control)	7	11,590	1,867	1	1	149
22	F_{10} (control)	21	18,674	1,400	1	0	NA
23	F_{10} (control)	12	11,616	1,671	1	1	353
24	F_{10} (control)	24	10,071	1,704	1	1	723
25	F_{10} (control)	3	13,867	0,994	1	1	138
26	F_{10} (control)	NA	NA	NA	NA	NA	NA
27	F_{10} (control)	NA	NA	NA	NA	NA	NA
28	F_{10} (control)	NA	NA	NA	NA	NA	NA
1	F_{10+400} (delay)	9	10,421	2,143	1	1	173
2	F_{10+400} (delay)	6	13,664	1,161	1	1	159
3	F_{10+400} (delay)	23	15,489	1,533	1	0	NA
4	F_{10+400} (delay)	1	14,009	0,067	0	NA	NA
5	F_{10+400} (delay)	NA	NA	NA	NA	NA	NA
6	F_{10+400} (delay)	2	17,184	0,133	0	NA	NA
7	F_{10+400} (delay)	NA	NA	NA	NA	NA	NA
8	F_{10+400} (delay)	25	13,484	1,667	1	0	NA
9	F_{10+400} (delay)	4	18,463	0,267	0	NA	NA

10	F_{10+400} (delay)	15	18,748	1,000	1	0	NA
11	F_{10+400} (delay)	28	24,037	1,867	1	0	NA
12	F_{10+400} (delay)	NA	NA	NA	NA	NA	NA
13	F_{10+400} (delay)	30	18,754	2,000	1	0	NA
14	F_{10+400} (delay)	7	14,622	0,467	1	0	NA
15	F_{10+400} (delay)	3	17,258	0,200	0	NA	NA
16	F_{10+400} (delay)	NA	NA	NA	NA	NA	NA
17	F_{10+400} (delay)	NA	NA	NA	NA	NA	NA
18	F_{10+400} (delay)	10	16,078	0,667	0	NA	NA
19	F_{10+400} (delay)	NA	NA	NA	NA	NA	NA
20	F_{10+400} (delay)	18	11,547	1,200	1	0	NA
21	F_{10+400} (delay)	1	16,163	0,067	0	NA	NA
22	F_{10+400} (delay)	25	18,582	1,667	1	0	NA
23	F_{10+400} (delay)	21	12,301	1,400	1	0	NA
24	F_{10+400} (delay)	NA	NA	NA	NA	NA	NA
25	F_{10+400} (delay)	3	16,498	0,849	1	1	142
26	F_{10+400} (delay)	1	14,701	0,233	0	NA	NA
27	F_{10+400} (delay)	NA	NA	NA	NA	NA	NA
28	F_{10+400} (delay)	3	19,041	0,200	0	NA	NA
1	F_{10+800} (delay)	25	16,412	1,667	1	0	NA
2	F_{10+800} (delay)	8	18,444	0,533	1	0	NA
3	F_{10+800} (delay)	22	20,803	1,467	1	0	NA
4	F_{10+800} (delay)	3	18,670	0,200	0	NA	NA
5	F_{10+800} (delay)	12	13,917	1,006	1	1	637
6	F_{10+800} (delay)	21	14,006	1,400	1	0	NA
7	F_{10+800} (delay)	15	20,282	1,000	0	NA	NA
8	F_{10+800} (delay)	4	12,534	0,267	0	NA	NA
9	F_{10+800} (delay)	9	17,461	0,600	0	NA	NA
10	F_{10+800} (delay)	2	14,226	0,133	0	NA	NA
11	F_{10+800} (delay)	24	19,142	1,600	0	NA	NA
12	F_{10+800} (delay)	3	13,659	0,773	1	1	64
13	F_{10+800} (delay)	8	17,100	1,569	1	1	210
14	F_{10+800} (delay)	3	21,036	0,200	1	0	NA
15	F_{10+800} (delay)	3	19,629	0,344	1	0	NA
16	F_{10+800} (delay)	NA	NA	NA	NA	NA	NA
17	F_{10+800} (delay)	NA	NA	NA	NA	NA	NA
18	F_{10+800} (delay)	1	16,215	0,067	0	NA	NA
19	F_{10+800} (delay)	NA	NA	NA	NA	NA	NA
20	F_{10+800} (delay)	6	15,878	0,400	1	0	NA
21	F_{10+800} (delay)	2	13,105	0,133	0	NA	NA
22	F_{10+800} (delay)	4	20,759	0,267	0	NA	NA
23	F_{10+800} (delay)	3	14,244	0,437	0	NA	NA

24	F_{10+800} (delay)	4	17,163	0,267	0	NA	NA
25	F_{10+800} (delay)	34	13,104	2,267	1	0	NA
26	F_{10+800} (delay)	NA	NA	NA	NA	NA	NA
27	F_{10+800} (delay)	11	15,865	1,262	1	0	NA
28	F_{10+800} (delay)	NA	NA	NA	NA	NA	NA
1	$F_{10+1500}$ (delay)	24	16,822	1,600	1	0	NA
2	$F_{10+1500}$ (delay)	5	15,529	0,375	1	0	NA
3	$F_{10+1500}$ (delay)	15	18,603	1,000	1	0	NA
4	$F_{10+1500}$ (delay)	8	18,418	0,584	0	NA	NA
5	$F_{10+1500}$ (delay)	NA	NA	NA	NA	NA	NA
6	$F_{10+1500}$ (delay)	21	12,924	1,400	1	0	NA
7	$F_{10+1500}$ (delay)	NA	NA	NA	NA	NA	NA
8	$F_{10+1500}$ (delay)	8	16,923	0,533	0	NA	NA
9	$F_{10+1500}$ (delay)	9	17,170	0,600	0	NA	NA
10	$F_{10+1500}$ (delay)	12	13,027	0,800	1	0	NA
11	$F_{10+1500}$ (delay)	7	22,128	0,657	1	1	546
12	$F_{10+1500}$ (delay)	NA	NA	NA	NA	NA	NA
13	$F_{10+1500}$ (delay)	11	16,102	0,733	1	0	NA
14	$F_{10+1500}$ (delay)	6	17,310	0,400	0	NA	NA
15	$F_{10+1500}$ (delay)	9	18,727	0,600	0	NA	NA
16	$F_{10+1500}$ (delay)	2	15,886	0,133	0	NA	NA
17	$F_{10+1500}$ (delay)	NA	NA	NA	NA	NA	NA
18	$F_{10+1500}$ (delay)	2	14,244	0,133	0	NA	NA
19	$F_{10+1500}$ (delay)	2	21,034	0,133	0	NA	NA
20	$F_{10+1500}$ (delay)	4	14,778	0,267	0	NA	NA
21	$F_{10+1500}$ (delay)	2	16,746	0,133	0	NA	NA
22	$F_{10+1500}$ (delay)	1	23,212	0,067	0	NA	NA
23	$F_{10+1500}$ (delay)	3	18,487	0,200	0	NA	NA
24	$F_{10+1500}$ (delay)	8	18,709	0,533	0	NA	NA
25	$F_{10+1500}$ (delay)	14	16,829	1,140	1	1	617
26	$F_{10+1500}$ (delay)	NA	NA	NA	NA	NA	NA
27	$F_{10+1500}$ (delay)	NA	NA	NA	NA	NA	NA
28	$F_{10+1500}$ (delay)	NA	NA	NA	NA	NA	NA
1	$F_{10+2000}$ (delay)	NA	NA	NA	NA	NA	NA
2	$F_{10+2000}$ (delay)	NA	NA	NA	NA	NA	NA
3	$F_{10+2000}$ (delay)	15	17,545	1,187	1	1	592
4	$F_{10+2000}$ (delay)	3	17,997	0,200	0	NA	NA
5	$F_{10+2000}$ (delay)	NA	NA	NA	NA	NA	NA
6	$F_{10+2000}$ (delay)	1	12,299	0,067	0	NA	NA
7	$F_{10+2000}$ (delay)	NA	NA	NA	NA	NA	NA
8	$F_{10+2000}$ (delay)	12	15,796	1,059	1	1	584
9	$F_{10+2000}$ (delay)	2	14,626	0,133	0	NA	NA

10	$F_{10+2000}$ (delay)	7	16,301	0,467	0	NA	NA
11	$F_{10+2000}$ (delay)	5	17,315	0,446	1	1	275
12	$F_{10+2000}$ (delay)	NA	NA	NA	NA	NA	NA
13	$F_{10+2000}$ (delay)	2	17,138	0,133	0	NA	NA
14	$F_{10+2000}$ (delay)	3	20,401	0,200	0	NA	NA
15	$F_{10+2000}$ (delay)	2	19,265	0,133	0	NA	NA
16	$F_{10+2000}$ (delay)	NA	NA	NA	NA	NA	NA
17	$F_{10+2000}$ (delay)	5	8,804	0,763	1	1	293
18	$F_{10+2000}$ (delay)	2	17,350	0,133	0	NA	NA
19	$F_{10+2000}$ (delay)	NA	NA	NA	NA	NA	NA
20	$F_{10+2000}$ (delay)	3	16,360	0,200	0	NA	NA
21	$F_{10+2000}$ (delay)	2	16,702	0,133	0	NA	NA
22	$F_{10+2000}$ (delay)	8	20,769	0,533	0	NA	NA
23	$F_{10+2000}$ (delay)	4	16,407	0,267	0	NA	NA
24	$F_{10+2000}$ (delay)	NA	NA	NA	NA	NA	NA
25	$F_{10+2000}$ (delay)	5	12,580	0,334	1	1	162
26	$F_{10+2000}$ (delay)	4	16,360	0,267	1	0	NA
27	$F_{10+2000}$ (delay)	NA	NA	NA	NA	NA	NA
28	$F_{10+2000}$ (delay)	2	12,877	0,133	0	NA	NA

S2 Table. Raw data from playback treatments used to assess whether males perceive female reply while calling.

male_identity	experiment	number of calls	average call duration [s]	calling rate [min^{-1}]	searching	locating	searching time [s]
1	F_5 (control)	21	7,801	1,858	1	1	541
2	F_5 (control)	2	20,304	0,133	0	NA	NA
3	F_5 (control)	33	16,002	2,200	1	0	NA
4	F_5 (control)	11	14,953	0,733	1	0	NA
5	F_5 (control)	NA	NA	NA	NA	NA	NA
6	F_5 (control)	25	11,836	3,080	1	1	407
7	F_5 (control)	8	15,732	0,533	0	NA	NA
8	F_5 (control)	39	17,283	2,600	1	0	NA
9	F_5 (control)	13	17,358	0,867	0	NA	NA
10	F_5 (control)	17	13,683	2,649	1	1	318
11	F_5 (control)	30	20,286	2,000	1	0	NA
12	F_5 (control)	NA	NA	NA	NA	NA	NA
13	F_5 (control)	22	13,758	2,463	1	1	390
14	F_5 (control)	38	10,930	3,262	1	1	623
15	F_5 (control)	40	13,737	2,667	1	0	NA
16	F_5 (control)	NA	NA	NA	NA	NA	NA
17	F_5 (control)	NA	NA	NA	NA	NA	NA
18	F_5 (control)	4	16,046	0,267	0	NA	NA
19	F_5 (control)	21	11,905	2,577	1	1	372
20	F_5 (control)	8	10,956	0,594	0	NA	NA
21	F_5 (control)	20	14,308	2,174	1	1	406
22	F_5 (control)	40	13,947	2,667	1	0	NA
23	F_5 (control)	24	12,451	1,600	1	0	NA
24	F_5 (control)	27	11,981	1,800	1	0	NA
25	F_5 (control)	40	11,116	3,196	1	1	666
26	F_5 (control)	NA	NA	NA	NA	NA	NA
27	F_5 (control)	30	15,036	2,000	1	0	NA
28	F_5 (control)	NA	NA	NA	NA	NA	NA
1	F_{5H} (hidden)	35	21,828	2,333	1	0	NA
2	F_{5H} (hidden)	2	24,844	0,133	0	NA	NA
3	F_{5H} (hidden)	19	21,003	1,541	1	1	573
4	F_{5H} (hidden)	2	10,695	0,284	0	NA	NA
5	F_{5H} (hidden)	29	18,861	2,112	1	1	749
6	F_{5H}	14	20,523	0,933	0	NA	NA

	(hidden)						
7	F_{5H} (hidden)	4	18,161	0,267	0	NA	NA
8	F_{5H} (hidden)	23	21,661	1,533	0	NA	NA
9	F_{5H} (hidden)	8	17,849	0,533	0	NA	NA
10	F_{5H} (hidden)	13	21,609	0,867	1	0	NA
11	F_{5H} (hidden)	1	21,290	0,067	0	NA	NA
12	F_{5H} (hidden)	NA	NA	NA	NA	NA	NA
13	F_{5H} (hidden)	35	20,336	2,333	1	0	NA
14	F_{5H} (hidden)	1	13,902	0,067	0	NA	NA
15	F_{5H} (hidden)	18	20,167	1,200	1	0	NA
16	F_{5H} (hidden)	NA	NA	NA	NA	NA	NA
17	F_{5H} (hidden)	NA	NA	NA	NA	NA	NA
18	F_{5H} (hidden)	1	15,919	0,067	0	NA	NA
19	F_{5H} (hidden)	3	20,089	0,200	0	NA	NA
20	F_{5H} (hidden)	28	18,508	2,565	1	0	NA
21	F_{5H} (hidden)	1	17,745	0,067	0	NA	NA
22	F_{5H} (hidden)	8	18,289	0,533	0	NA	NA
23	F_{5H} (hidden)	18	20,627	1,200	1	0	NA
24	F_{5H} (hidden)	7	19,806	0,467	0	NA	NA
25	F_{5H} (hidden)	26	20,175	1,733	1	0	NA
26	F_{5H} (hidden)	NA	NA	NA	NA	NA	NA
27	F_{5H} (hidden)	NA	NA	NA	NA	NA	NA
28	F_{5H} (hidden)	NA	NA	NA	NA	NA	NA
1	F_0 (control)	1	12,159	0,067	0	NA	NA
2	F_0 (control)	NA	NA	NA	NA	NA	NA
3	F_0 (control)	36	21,263	2,400	1	0	NA
4	F_0 (control)	1	16,109	0,067	0	NA	NA
5	F_0 (control)	NA	NA	NA	NA	NA	NA
6	F_0 (control)	27	20,075	1,800	1	0	NA
7	F_0 (control)	NA	NA	NA	NA	NA	NA

8	F_0 (control)	38	19,221	2,533	1	0	NA
9	F_0 (control)	6	16,607	0,400	0	NA	NA
10	F_0 (control)	3	17,976	0,277	1	1	618
11	F_0 (control)	36	23,595	2,400	0	NA	NA
12	F_0 (control)	NA	NA	NA	NA	NA	NA
13	F_0 (control)	3	19,728	0,200	1	0	NA
14	F_0 (control)	3	19,761	0,200	0	NA	NA
15	F_0 (control)	34	19,076	2,267	1	0	NA
16	F_0 (control)	NA	NA	NA	NA	NA	NA
17	F_0 (control)	NA	NA	NA	NA	NA	NA
18	F_0 (control)	3	18,621	0,200	0	NA	NA
19	F_0 (control)	NA	NA	NA	NA	NA	NA
20	F_0 (control)	19	20,179	1,267	0	NA	NA
21	F_0 (control)	5	21,146	0,333	1	0	NA
22	F_0 (control)	14	12,766	0,933	1	0	NA
23	F_0 (control)	3	19,218	0,200	0	NA	NA
24	F_0 (control)	NA	NA	NA	NA	NA	NA
25	F_0 (control)	20	22,142	1,333	1	0	NA
26	F_0 (control)	4	16,857	0,647	0	NA	NA
27	F_0 (control)	NA	NA	NA	NA	NA	NA
28	F_0 (control)	NA	NA	NA	NA	NA	NA
