# Supplementary material for: The Effect of Timing of Female Vibrational Reply on Male Signalling and Searching Behaviour in the Leafhopper Aphrodes makarovi
Source: PLoS One. 2015 Oct 21;10(10):e0139020. doi: 10.1371/journal.pone.0139020 (PMC4619402; doi:10.1371/journal.pone.0139020)
Supplement: S2 Table — The table summarizes signalling and searching behavioural parameters for each male scored in each treatment. (PDF) [file pone.0139020.s003.pdf]

S3 Table. Raw data from playback treatments used to assess whether males perceive female reply while calling. The table summarizes signalling and searching behavioural parameters for each male scored in each treatment.

male_identity	experiment	number of calls	average call duration [s]	calling rate [min^{-1}]	searching	locating	searching time [s]
1	F_5 (control)	21	7,801	1,858	1	1	541
2	F_5 (control)	2	20,304	0,133	0	NA	NA
3	F_5 (control)	33	16,002	2,200	1	0	NA
4	F_5 (control)	11	14,953	0,733	1	0	NA
5	F_5 (control)	NA	NA	NA	NA	NA	NA
6	F_5 (control)	25	11,836	3,080	1	1	407
7	F_5 (control)	8	15,732	0,533	0	NA	NA
8	F_5 (control)	39	17,283	2,600	1	0	NA
9	F_5 (control)	13	17,358	0,867	0	NA	NA
10	F_5 (control)	17	13,683	2,649	1	1	318
11	F_5 (control)	30	20,286	2,000	1	0	NA
12	F_5 (control)	NA	NA	NA	NA	NA	NA
13	F_5 (control)	22	13,758	2,463	1	1	390
14	F_5 (control)	38	10,930	3,262	1	1	623
15	F_5 (control)	40	13,737	2,667	1	0	NA
16	F_5 (control)	NA	NA	NA	NA	NA	NA
17	F_5 (control)	NA	NA	NA	NA	NA	NA
18	F_5 (control)	4	16,046	0,267	0	NA	NA
19	F_5 (control)	21	11,905	2,577	1	1	372
20	F_5 (control)	8	10,956	0,594	0	NA	NA
21	F_5 (control)	20	14,308	2,174	1	1	406
22	F_5 (control)	40	13,947	2,667	1	0	NA
23	F_5 (control)	24	12,451	1,600	1	0	NA
24	F_5 (control)	27	11,981	1,800	1	0	NA
25	F_5 (control)	40	11,116	3,196	1	1	666
26	F_5 (control)	NA	NA	NA	NA	NA	NA
27	F_5 (control)	30	15,036	2,000	1	0	NA
28	F_5 (control)	NA	NA	NA	NA	NA	NA
1	F_{5H} (hidden)	35	21,828	2,333	1	0	NA
2	F_{5H} (hidden)	2	24,844	0,133	0	NA	NA
3	F_{5H} (hidden)	19	21,003	1,541	1	1	573
4	F_{5H} (hidden)	2	10,695	0,284	0	NA	NA
5	F_{5H} (hidden)	29	18,861	2,112	1	1	749
6	F_{5H} (hidden)	14	20,523	0,933	0	NA	NA

7	F_{5H} (hidden)	4	18,161	0,267	0	NA	NA
8	F_{5H} (hidden)	23	21,661	1,533	0	NA	NA
9	F_{5H} (hidden)	8	17,849	0,533	0	NA	NA
10	F_{5H} (hidden)	13	21,609	0,867	1	0	NA
11	F_{5H} (hidden)	1	21,290	0,067	0	NA	NA
12	F_{5H} (hidden)	NA	NA	NA	NA	NA	NA
13	F_{5H} (hidden)	35	20,336	2,333	1	0	NA
14	F_{5H} (hidden)	1	13,902	0,067	0	NA	NA
15	F_{5H} (hidden)	18	20,167	1,200	1	0	NA
16	F_{5H} (hidden)	NA	NA	NA	NA	NA	NA
17	F_{5H} (hidden)	NA	NA	NA	NA	NA	NA
18	F_{5H} (hidden)	1	15,919	0,067	0	NA	NA
19	F_{5H} (hidden)	3	20,089	0,200	0	NA	NA
20	F_{5H} (hidden)	28	18,508	2,565	1	0	NA
21	F_{5H} (hidden)	1	17,745	0,067	0	NA	NA
22	F_{5H} (hidden)	8	18,289	0,533	0	NA	NA
23	F_{5H} (hidden)	18	20,627	1,200	1	0	NA
24	F_{5H} (hidden)	7	19,806	0,467	0	NA	NA
25	F_{5H} (hidden)	26	20,175	1,733	1	0	NA
26	F_{5H} (hidden)	NA	NA	NA	NA	NA	NA
27	F_{5H} (hidden)	NA	NA	NA	NA	NA	NA
28	F_{5H} (hidden)	NA	NA	NA	NA	NA	NA
1	F_0 (control)	1	12,159	0,067	0	NA	NA
2	F_0 (control)	NA	NA	NA	NA	NA	NA
3	F_0 (control)	36	21,263	2,400	1	0	NA
4	F_0 (control)	1	16,109	0,067	0	NA	NA
5	F_0 (control)	NA	NA	NA	NA	NA	NA
6	F_0 (control)	27	20,075	1,800	1	0	NA
7	F_0 (control)	NA	NA	NA	NA	NA	NA
8	F_0 (control)	38	19,221	2,533	1	0	NA

9	F_0 (control)	6	16,607	0,400	0	NA	NA
10	F_0 (control)	3	17,976	0,277	1	1	618
11	F_0 (control)	36	23,595	2,400	0	NA	NA
12	F_0 (control)	NA	NA	NA	NA	NA	NA
13	F_0 (control)	3	19,728	0,200	1	0	NA
14	F_0 (control)	3	19,761	0,200	0	NA	NA
15	F_0 (control)	34	19,076	2,267	1	0	NA
16	F_0 (control)	NA	NA	NA	NA	NA	NA
17	F_0 (control)	NA	NA	NA	NA	NA	NA
18	F_0 (control)	3	18,621	0,200	0	NA	NA
19	F_0 (control)	NA	NA	NA	NA	NA	NA
20	F_0 (control)	19	20,179	1,267	0	NA	NA
21	F_0 (control)	5	21,146	0,333	1	0	NA
22	F_0 (control)	14	12,766	0,933	1	0	NA
23	F_0 (control)	3	19,218	0,200	0	NA	NA
24	F_0 (control)	NA	NA	NA	NA	NA	NA
25	F_0 (control)	20	22,142	1,333	1	0	NA
26	F_0 (control)	4	16,857	0,647	0	NA	NA
27	F_0 (control)	NA	NA	NA	NA	NA	NA
28	F_0 (control)	NA	NA	NA	NA	NA	NA
